# Walter Reed Memorial Association

**Published:** 1904-08

**Authors:** C. DeWitt

**Affiliations:** 1707 21st Street, N. W., Washington, D. C.


### CORRESPONDENCE.

### Walter Reed Memorial Association.

**$25,000 to be Raised. — Subscriptions Solicited.**

*Editor Buffalo Medical Journal:*

Sir.—I am sure you will agree with us that the scientific work of the late Major Walter Reed, U. S. A., which has led to such beneficent results, should receive some special recognition. With this object in view the "Walter Reed Memorial Association" has been incorporated under the laws of the District of Columbia. After careful consideration we have decided that this is the most practical manner of securing a responsible board of trustees to take charge of the funds subscribed for a memorial to Major

Reed, and to attend to the execution of the project when the necessary money has been received. The American Medical Association and the Association for the Advancement of Science have appointed committees which will no doubt coöperate with us, and subscriptions to the fund may be handed to any member of these committees, or they may be sent to Mr. Chas. J. Bell, president of the American Security and Trust Company, Washington, D. C., who has consented to act as treasurer. It is thought that at least $25,000 should be raised, and it is probable that the exact character of the memorial will not be determined until a considerable portion of this amount is in the hands of the treasurer. While we feel that the non-medical public should contribute a considerable portion of the amount required, we must depend mainly upon the physicians to arouse interest in the project by pointing out the important results of Major Reed's yellow fever investigations.

Will you not aid us in this way and also by any personal contribution you may be willing to make? Correspondence may be addressed to the secretary.

By order of the executive committee.

C. DEWITT, *Secretary*,
1707 21st Street, N. W.

WASHINGTON, D. C., July, 1904.

